# Transdermal delivery of colchicine using dissolvable microneedle arrays for the treatment of acute gout in a rat model

**DOI:** 10.1080/10717544.2022.2122632

**Published:** 2022-09-13

**Authors:** Yang Liu, Xiaoruo Zhu, Shiliang Ji, Zhen Huang, Yuhui Zang, Ying Ding, Junfeng Zhang, Zhi Ding

**Affiliations:** a State Key Laboratory of Pharmaceutical Biotechnology, School of Life Sciences, Nanjing University, Nanjing, China; b Department of Pharmacy, Suzhou Science & Technology Town Hospital, Gusu School, Nanjing Medical University, Suzhou, China; c Department of Anesthesiology, The Second Affiliated Hospital of Nanjing Medical University, Nanjing, China; d Engineering Research Center of Protein and Peptide Medicine, Ministry of Education, Nanjing, China; e Changzhou High-Tech Research Institute of Nanjing University, Changzhou, China

**Keywords:** Colchicine, soluble microneedles, transdermal drug delivery, acute gout, rats

## Abstract

Colchicine (Col) is used to prevent and treat acute gout flare; however, its therapeutic use is strictly limited owing to severe gastrointestinal side effects after oral administration. Therefore, we developed a dissolvable Col-loaded microneedle (MN) with hyaluronic acid to deliver Col *via* the transdermal route. We studied the preparation, mechanical properties, skin insertion, skin irritation, drug content, and transdermal release of the Col-loaded MN. The pharmacokinetics of Col after Col-loaded MN application were compared with those of Col solution gavage over 24 h. Knee joint edema evaluation and the hindfoot mechanical threshold test were conducted to determine the pharmacodynamic profile. The dissolvable Col-loaded MN possessed sufficient mechanical strength to penetrate the skin and release the loaded drug. No skin irritation was observed for 3 days after application. We found that 3.36-fold more Col contained in MNs was delivered through the skin compared with that in gel *in vitro*, and moderate relative bioavailability *in vivo*. The Col-loaded MN significantly relieved swollen knee joints and mechanical hypernociception in an acute gout model in rats. The dissolvable Col-loaded MN array reduced inflammation and pain via topical administration when acute gout occurred. Reducing the gastrointestinal side effects of Col-loaded MNs is expected to optimize the therapeutic effects of Col and improve patient compliance.

## Introduction

Gout, a common chronic disease that usually manifests as acute or chronic joint inflammation, is a type of inflammatory arthritis caused by increased urate concentrations and the deposition of monosodium urate (MSU) crystals (Harris et al., 1999; Abrahams, 2015; Dalbeth et al., 2021). Gout and hyperuricemia result in metabolic comorbidities, such as hypertension, hyperlipidemia, atherosclerosis, nephropathy, and obesity (Dalbeth et al., 2019). MSU crystals interact with resident macrophages, resulting in the activation of the NOD-like receptor family pyrin domain-containing protein 3 inflammasome and subsequent release of active interleukin-1β, which plays critical roles at the beginning of acute gout flares by initiating the production and secretion of pro-inflammatory mediators (Martinon, 2010).

Acute gout therapy includes the effective control of the inflammatory response, the principal objective of which is the rapid and safe resolution of pain and acute flares (Sidari & Hill, 2018); non-steroidal anti-inflammatory drugs, such as colchicine (Col) and corticosteroids, are recommended medicines (Tang et al., 2020). Col, a tricyclic alkaloid that exerts inhibitory effects on several inflammatory cascade pathways in leukocytes and inhibits the phagocytosis of MSU crystals by neutrophils, has been widely used for acute gout flare prophylaxis and treatment at a daily dose of 1.2–1.8 mg (Slobodnick et al., 2018). However, as Col is generally administered orally, gastrointestinal toxicity is the most common dose-limiting side effect (Pascart & Richette, 2018). Approximately 80% of patients experience severe symptoms of diarrhea, nausea, and vomiting, which decreases patient compliance and limits the clinical application of Col (Putterman et al., 1991; Harris et al., 1999).

The skin is composed of epidermis, dermis, and subcutaneous tissues (Yang et al., 2021). Abundant blood and lymphatic vessels in the dermis and subcutaneous tissue are linked with systemic circulation, thus, the unique physiological structure of the skin provides an easily accessible site optimal for drug delivery (Kanitakis, 2002). However, the epidermis, especially the *stratum corneum* (SC), forms a skin permeation barrier, which prevents drug molecules from entering systemic circulation (Chen, 2018; Yang et al., 2021).

Compared with oral administration and injection, transdermal drug delivery can achieve drug release in a minimally invasive manner (Puri et al., 2021). Drugs can be delivered through the skin in the form of gels, creams, ointments, and patches (Cheung & Das, 2016; Tucak et al., 2020). Overcoming the skin barrier function and increasing skin permeability are key issues for transdermal drug delivery. Zhang *et al*. prepared modified Col ethosomes, which showed an adequate transdermal delivery efficiency, with a time to maximum plasma concentration (*T_ma_*_x_) as slow as 3 h (Zhang et al., 2019). Additionally, Abdulbaqi *et al*. prepared Col transethosomal gels that could continuously release Col but were not suitable for storage at room temperature because of the particle instability and significant drug content leakage after one-month storage (Abdulbaqi et al., 2018). Moreover, physical and chemical methods, such as iontophoresis (Singh & Roberts, 1989), low-frequency ultrasound (Kushner et al., 2007; Park et al., 2014), puncture (Yang et al., 2018), electroporation (Ita, 2016), laser (Lee et al., 2001), and chemical penetration methods (Kushner et al., 2007), can disturb the structure of the SC and increase drug permeability. However, these strategies may cause skin damage and require additional external instruments.

Microneedles (MNs), which have a needle length of less than 1 mm, can enhance the ability of a drug to cross the skin with little pain or bleeding (Tucak et al., 2020). MNs were designed to facilitate transdermal drug delivery; however, today, their use has progressed to generation of smart MNs for therapy and diagnosis (Hassan et al., 2017; Zhang et al., 2020), including biomarker detection and transdermal drug delivery (Amani et al., 2021). MNs can facilitate effective delivery of drugs, including those for diabetes (Jin et al., 2018), vaccinations (Rodgers et al., 2018), and dermatological conditions (Du et al., 2019; Sabri et al., 2019; Chen et al., 2020), which include insulin, vaccines, and various high and low molecular weight compounds. In addition, the use of MNs in rapid diagnostics enables the detection of biomarkers such as those for malaria (Jiang & Lillehoj, 2020) in dermal interstitial fluid and detection of pathogenic bacteria in food (Kim et al., 2021). Among the several types of MNs, dissolvable MNs are composed of a soluble matrix of biocompatible polysaccharides or polymers, such as sodium hyaluronate (HA) (Qiu et al., 2016), a biodegradable and biocompatible polymer with minimal immunogenicity, widely used in the cosmetic industry. HA has high mechanical strength when dehydrated and adsorbs water quickly (Yang et al., 2021). Huisuk *et al* prepared lidocaine-loaded dissolving MNs, and demonstrated that MN application for 10 min could achieve almost the same anesthetic effect as cream formulation application for 60 min, suggesting that dissolving MNs have a rapid onset of action (Yang et al., 2020). When applied, MNs come into contact with the skin tissue fluid and dissolve at an appropriate speed, releasing the loaded drug onto the skin without producing any sharp, biologically contaminated, and non-degradable waste (McGrath et al., 2011).

**Figure 1.**
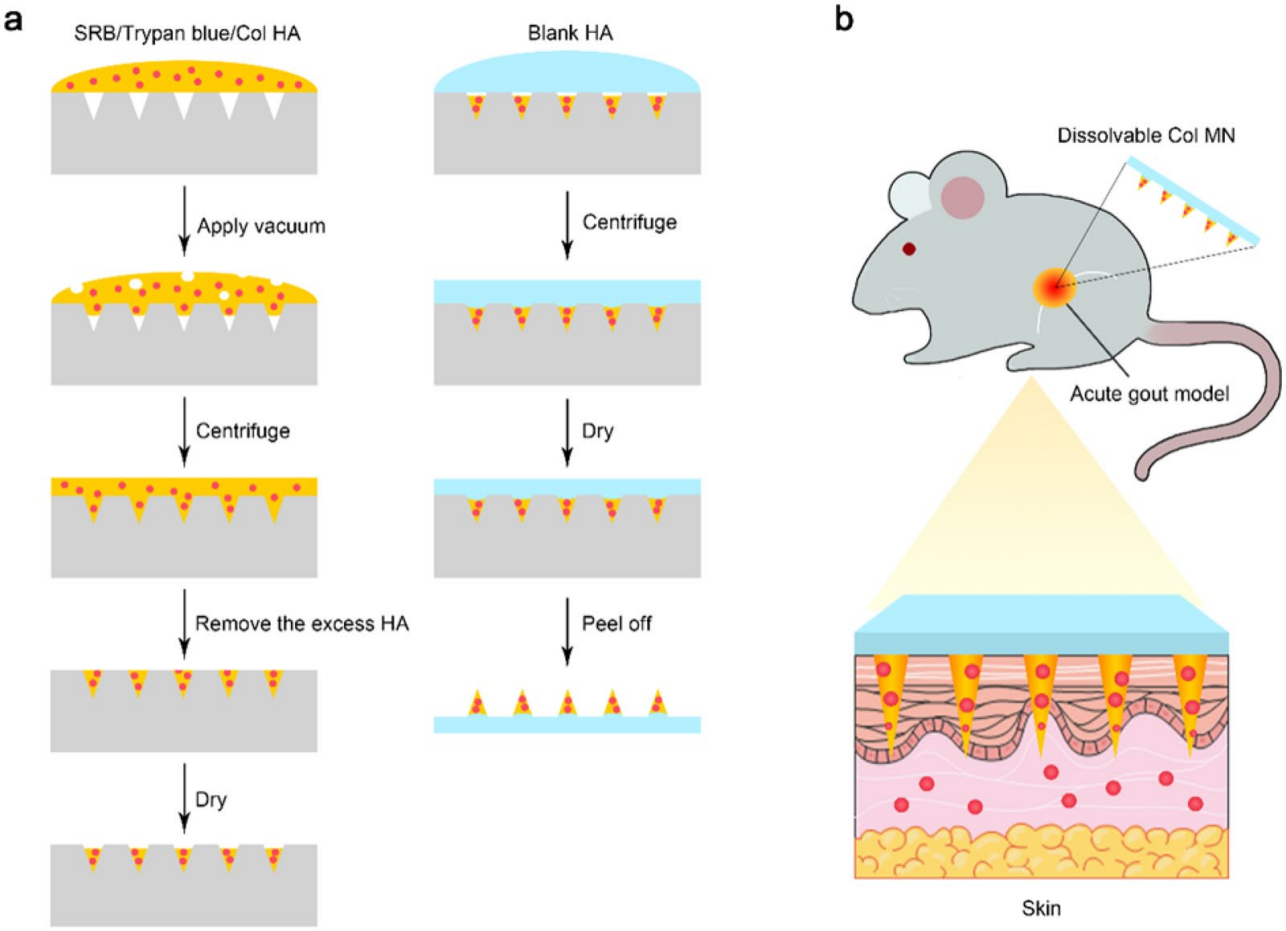
Fabrication and application of Col-loaded MNs. (a) Dissolvable MNs were prepared by a mold casting method using HA (w/o certain substances) addition, vacuum centrifugation, and a drying procedure. (b) Col-loaded MN array was locally applied to the skin of rats with acute gout to achieve transdermal Col delivery with minimal invasion. Abbreviations: SRB: sulforhodamine B; Col, colchicine; MN, microneedle; HA, sodium hyaluronate.

To the best of our knowledge, there has not been a study on the transdermal delivery of Col in the form of a dissolving MN. In this study, we developed a dissolvable Col-loaded MN to deliver Col locally via transdermal administration (Figure 1). The purpose of our study was to achieve comparable anti-inflammatory and pain control effects of Col at lower doses with fewer side effects when administered via topical administration. We hope that this Col-loaded MN strategy can help increase acute-gout patients’ compliance to drug intake in the future.

## Materials and methods

### Materials

HA (molecular weight [MW]: 8510 Da, 340 kDa, and 1350 kDa) was purchased from Bloomage Biotechnology Co., Ltd. (Jinan, China). Col was purchased from Aladdin Co., Ltd. (Shanghai, China), and the Col standard was obtained from Meilunbio (Dalian, China). Uric acid was purchased from Sigma-Aldrich Inc. (Shanghai, China). Trypan blue was purchased from Shanghai Zeye Biotechnology Co., Ltd. (Shanghai, China). Sulforhodamine B (SRB) was purchased from Macklin Biochemical Co., Ltd. (Shanghai, China). Optimal cutting temperature (OCT)-freeze medium was obtained from Sakura Finetek Japan Co., Ltd. (Tokyo, Japan). All other chemicals used were of analytical grade, and all solutions were prepared using double-distilled water.

### Animals

Standardized and specific-pathogen-free (SPF) grade male Wistar rats (weight 160–200 g, 5–6 weeks old at the beginning of the experiments) were purchased from GemPharmaTech Co., Ltd. (Nanjing, China), and SPF male and female BALB/c mice (weight 20–25 g, 6–8 weeks old at the beginning of the experiments) were obtained from the Laboratory Animal Center of Nanjing Medical University (Nanjing, China). All animals were acclimatized for 1 week before further experiments and maintained under standardized pathogen-free conditions at the Laboratory Animal Center of the State Key Laboratory of Pharmaceutical Biotechnology, Nanjing University. All animals were raised at constant temperature (22 ± 1 °C) and humidity (55 ± 10%) with a 12 h light/dark cycle and free access to food and water. Animal experiments were approved by the Animal Welfare and Ethical Review Committee of Nanjing University (IACUC-2012002) and strictly performed in compliance with the Regulations of Experimental Animal Administration issued by the State Committee of Science and Technology of the People’s Republic of China. All animals were randomly allocated to the study groups. Every effort was made to minimize animal suffering and reduce the number of animals used in the experiment.

### Preparation and characterization of colchicine-loaded microneedle

The dissolvable Col-loaded MN array was fabricated using the mold casting method (Figure 1a). HA powder with or without Col was dissolved in degassed double-distilled water. Under magnetic stirring at 300 rpm for 7 h, polymer solutions of blank HA (40 mg/mL) or Col HA solutions (30 mg/mL + 40 mg/mL) were formed. Col HA solution (500 μL) was pipetted evenly in the mold and placed in a desiccator under vacuum for 30 min. The mold was then centrifuged at 1811 *×* *g* for 20 min to make the solution fully fill the microcavities. Subsequently, 300 μL of Col HA solution was pipetted evenly onto the mold and vacuumed for 30 min, and then centrifuged at 1811 × *g* for 10 min. The excess Col HA solution on the mold surface was removed and centrifuged at 1811 × *g* for 10 min. After drying in a vacuum incubator at 45 °C for 1 h, the blank HA solution was pipetted onto the mold and centrifuged at 1811 × *g* for 10 min to fabricate the plate layer. The MN was completely desiccated for at least 9 h at 45 °C, peeled off from the mold, attached to an adhesive patch, and sealed in a dry environment at RT (18–25 °C) for further analysis.

The morphology and integrity of the MNs were examined using a stereomicroscope (DS-Fi2, Nikon, Tokyo, Japan). To visualize the distribution of the loaded hydrophilic substance, trypan blue was loaded into the MN instead of Col using the same procedure.

### Mechanical strength measurement

The mechanical strength of the soluble Col-loaded MN was determined using a displacement force tester (HLX-S, Haibao, Wenzhou, China), during which the MN patch was compressed in the vertical direction between parallel metal plates. The displacement force tester is composed of a digital display push-pull gauge and an up-swing test bench, of which the rocker can be turned to move the mobile cylindrical force-measuring probe vertically downwards. The 10 × 10 microneedle array patches were made of HA of different MWs (8510 Da, 340 kDa, and 1350 kDa) and were stuck horizontally on the flat rigid base of the tester with double-sided tape, directly below the probe. When the probe touched the uppermost point of the microneedle tip, the displacement was recorded as 0 mm. The speed of the probe toward the MN was 0.02 mm/s. The force and displacement were recorded until the probe reached a pre-set distance of 0.6 mm.

### Skin penetration study

The SRB-loaded MNs were sterilized under UV light for 15 min. The dorsal skin of the rats was shaved, and the SRB-loaded MN array was applied to the skin of the rats for 2 min. Then, the rats were euthanized by cervical dislocation, the treated skin (∼2 × 2 cm^2^) was dissected and the subcutaneous tissue was removed. At 0, 0.5, 1, 1.5, and 2 h, an upright fluorescence microscope (TE2000-U, Nikon, Tokyo, Japan) was used to image and evaluate SRB diffusion and MN penetration of the skin.

In another experiment, the treated skin sample was frozen and sectioned for fluorescence analysis after applying the SRB-loaded MNs for 2 min. Approximately 1 × 1 cm^2^ of the tissue was obtained, frozen in OCT compound, and sectioned. The sections were fixed with 4% paraformaldehyde for 10 min and imaged using a laser scanning fluorescence microscope (Ti-C2, Nikon, Tokyo, Japan).

### Drug loading of colchicine-loaded microneedle

To determine the Col content in the MN, the same batch of MNs was dissolved in 1 mL of 1× PBS (pH 7.4) immediately after the preparation of the Col-loaded MN. After incubation at RT (18–25 °C) on a shaker for 2 h, a uniform solution was obtained and filtered through 0.22 μm filters. The filtrate was measured using high-performance liquid chromatography (HPLC; 1260, Agilent, Santa Clara, CA, USA), and the content of Col in the MN array was calculated according to the standard curve (Figure S1).

### Skin irritation test

After different treatments, erythema and edema on the shaved skin of healthy mice without scratches or wounds were observed. The mice were randomly divided into three groups: (1) blank MN group, (2) 5% sodium dodecyl sulfate (SDS) group, and (3) Col-loaded MN group. The skin on the left of the dorsal midline, deemed as a blank control, received no intervention except for hair removal. The skin on the right of the midline of the three groups received 5% SDS or MNs for 1 h per day for 3 consecutive days. On the fourth day, the untreated and treated skin sites were dissected and stained using a standard hematoxylin and eosin staining procedure.

### *In vitro* skin permeation study of colchicine-loaded microneedles

For the *in vitro* permeation study, MN patches loaded with Col were applied to the skin of the knee joints of rats, and 1× PBS (pH 7.4) was used as the receiver compartment medium. Specifically, three pieces of skin (1.5 × 1.5 cm^2^) were removed and cleaned with normal saline. Col-loaded MNs were applied to the rat skin for 2 min. For comparison, a homogenous gel with the same amount of Col was prepared with HA and applied to the rat skin, defined as the Col-L gel group. The skin was placed between the donor chamber and receiving cell in a Franz diffusion cell system (Xinzhou TP-6, Tianjin, China). Then, 4 mL prewarmed PBS (33.5 °C) and a magnetic stirrer were added in each receiving cell. To ensure that the solution infiltrated the peeling surface of the rat skin, possible air bubbles were carefully removed when loading the rat skin. The receiving cells were kept in a water bath (350 rpm) at 33.5 °C and away from light during the whole process. At pre-set intervals of 0.5, 1, 2, 4, 6, 8, and 24 h, 200 μL aliquots of sample were removed from the receiving cell, which was immediately refilled with 200 μL of 1× PBS solution. Finally, the obtained samples were used to determine the content of Col by HPLC, and a calibration curve was used to calculate the cumulative permeation volume.

### Colchicine pharmacokinetics after release from microneedle *in vivo*

The *in vivo* pharmacokinetic study was performed in back-shaved Wistar rats after 12 h of fasting. The rats were randomly divided into two groups: (1) Col gavage group, in which each rat was gavaged with 1 mL of Col (0.05 mg/mL) normal saline solution; (2) Col-loaded MN group, in which Col-loaded MNs were inserted into the back skin of rats.

Blood samples (∼200 μL) were collected from the retro-orbital vein into heparinized Eppendorf tubes at predetermined time intervals: (1) 0.25, 0.5, 1, 2, 4, 6, 8, 12, and 24 h after Col solution (*i.g.*) application; (2) 0.08, 0.25, 0.5, 1, 3, 6, 12, and 24 h after MN application. The clear plasma was separated by centrifugation at 2400 × *g* for 10 min at 4 °C. The plasma samples were vortex-mixed with four times the volume of methanol for 5 min, shaken for 5 min, and centrifuged at 13,800 × *g* for 10 min at 4 °C. The supernatant was then concentrated with a nitrogen concentrator (JXDC-400, JingXin, Shanghai, China) and resuspended in 60 μL of 50% acetonitrile aqueous solution. Another supernatant was obtained by centrifugation at 13,800 × *g* for 10 min at 4 °C, and liquid chromatography-tandem mass spectrometry (QTRAP 6500, AB Sciex, Framingham, MA, USA) was used for the determination of Col in the samples (details provided in Supplementary Materials). The relative bioavailability was calculated using the following formula (1).

(1) $$\frac{\text{AUC}\text{T×DR}}{\text{AUC}\text{R×DT}}$$

### Pharmacodynamic study of colchicine-loaded microneedle

MSU crystals were prepared by the crystallization of a supersaturated solution, as described previously (Nishimura et al., 1997). Briefly, 1 g of uric acid was dissolved in 200 mL of 1 M NaOH. The solution was heated to 60 °C, the pH was adjusted to 8.5 with HCl, and the solution was cooled down to crystallize at 4 °C for 24 h. The crystals were harvested, sterilized at 180 °C for 2 h, and suspended in sterile 1× PBS at 25 mg/mL. The rats were anesthetized with pentobarbital sodium (40 mg/kg, *i.p.*). Approximately 50 μL of the sterile suspension of MSU was injected into the left knee joint of rats through a 21-gauge needle (Coderre & Wall, 1988).

The rats were randomly divided into five groups: (1) blank MN group; (2) Col-L gel group; (3) Col-H gel group, in which gel was made of HA and Col (40 mg/mL) and applied at the dosage of 30 μL/rat; (4) Col gavage group (0.3 mg/rat); and (5) Col-loaded MN group. Knee edema and mechanical hyperalgesia were assessed before MSU injection (baseline), 12–24 h after MSU injection, and after the application of different treatments.

The formation of edema was assessed by measuring the knee diameters before MSU injection and 0, 1, 2, 3, 4, and 5 h after treatment. The mechanical paw withdrawal threshold (PWT) was evaluated using von Frey monofilaments (Dong et al., 2008). Rats were individually placed in transparent acrylic cube chambers on an elevated metal mesh and habituated for at least 30 min before the tests. An ascending series (0.008–300 g) of calibrated von Frey filaments (BW806, Shanghai Bio-will, Shanghai, China) was applied to the central area of the hind paw, lasting for 2–3 s at 1–2 min intervals. Brisk withdrawal or licking was recorded as a positive response.

### Statistical analysis

Unless otherwise stated, data are presented as mean ± standard error (SEM), and were analyzed using GraphPad Prism 7.0 (San Diego, CA, USA) with a two-sided *t*-test or ANOVA with Bonferroni’s post-hoc test. Statistical significance was set at *p*  <  .05.

## Results

### Fabrication and characterization of colchicine-loaded microneedle

**Figure 2.**
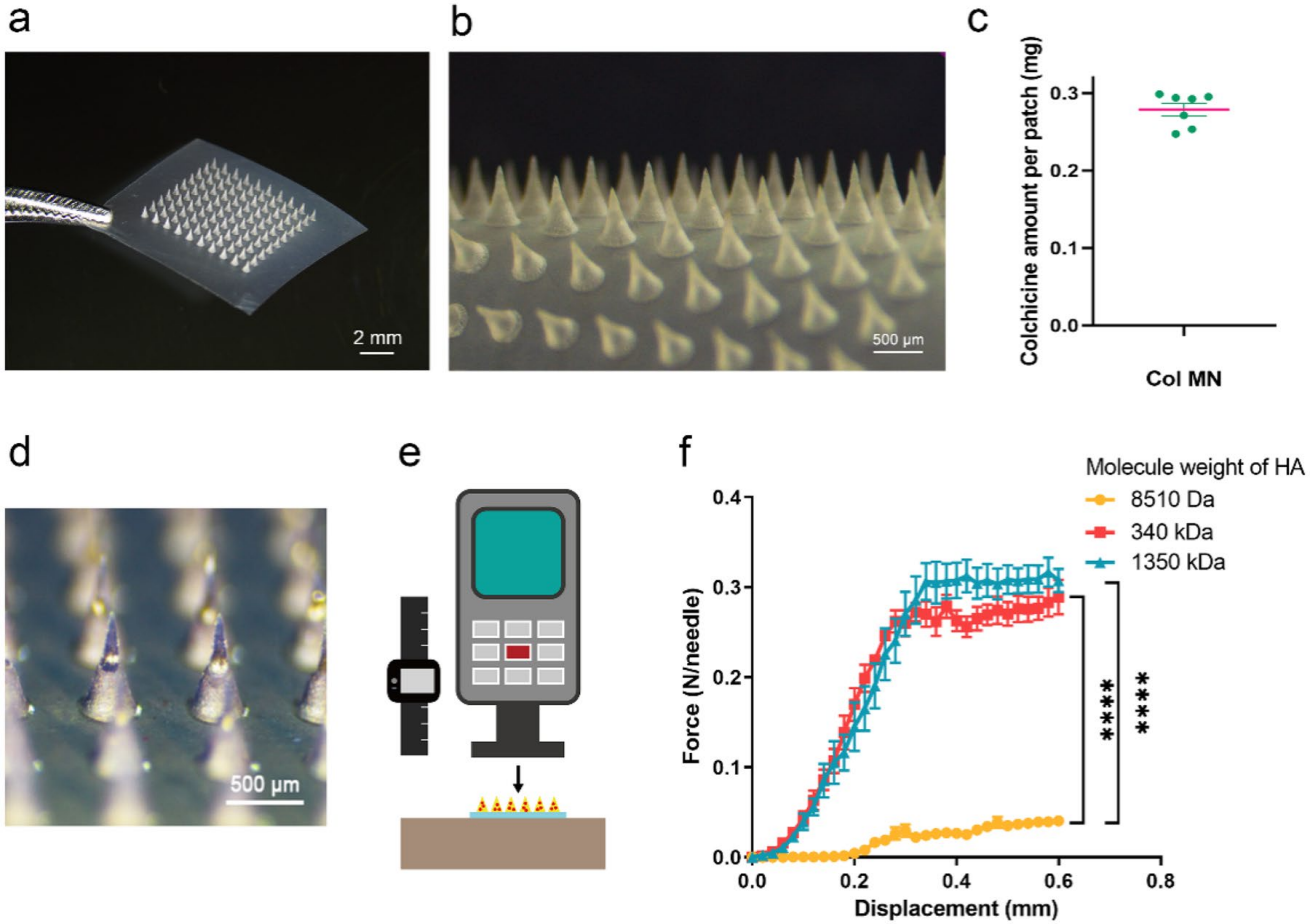
Characterization of dissolvable colchicine-loaded microneedle. (a and b) Stereomicroscopy of 10 × 10 Col-loaded MN array. (c) Drug content of 10 × 10 Col-loaded MN array; *n* = 7. (d) Stereomicroscopy of trypan blue MN array. (e) Diagram of displacement force tester, which applied vertical force to the MN array with increasing displacement. (f) The force displacement curve of the mechanical strength of MNs prepared by HA of different molecular weights; the abscissa is the moving distance of the tensimeter probe and the ordinate is the force exerted on each needle of the MN array; 8510 Da group, *n* = 3; 340 kDa group, *n* = 4; 1350 kDa group, *n* = 5. **** indicates statistical significance at *p*  <  .0001. Abbreviations: Col, colchicine; MN, microneedle; HA, sodium hyaluronate.

Dissolvable Col-loaded MNs were prepared with HA and observed using a stereomicroscope (Figure 2a). The MN array was composed of 10 × 10 conical needles, with an MN length of 600 μm, base diameter of 400 μm, and tip distance of 700 μm (Figure 2b). The average amount of Col loaded in the 10 × 10 MN array was 279 ± 8.14 µg (Figure 2c).

To visualize drug distribution more clearly and intuitively, trypan blue was loaded into the MNs instead of Col (Figure 2d). MNs had a consistent shape and spacing, and the substance contained in the MNs was more concentrated on the tips of the needles, facilitating its rapid release.

To investigate whether Col-loaded MNs can pierce the skin effectively, 10 × 10 MN arrays made of HA with different MWs were tested for axial strength (Figure 2e). The force displacement curve showed a continuous increase in pressure with displacement, and the MN only bent, without breaking, under the increasing pressure (Figure 2f). When the displacement was 0.6 mm, the maximum bearing force of the 8510 Da group (0.041 ± 0.004 N/needle) was significantly lower than that of the 340 kDa and 1350 kDa groups (0.289 ± 0.019 N/needle and 0.307 ± 0.013 N/needle, respectively) (*p*  <  .0001). As the gel made of HA (1350 kDa) was too viscous for preparation and retarded MN dissolution and Col release, HA (340 kDa) was chosen for follow-up experiments.

### *In vitro* skin insertion of dissolvable fluorescent microneedle

**Figure 3.**
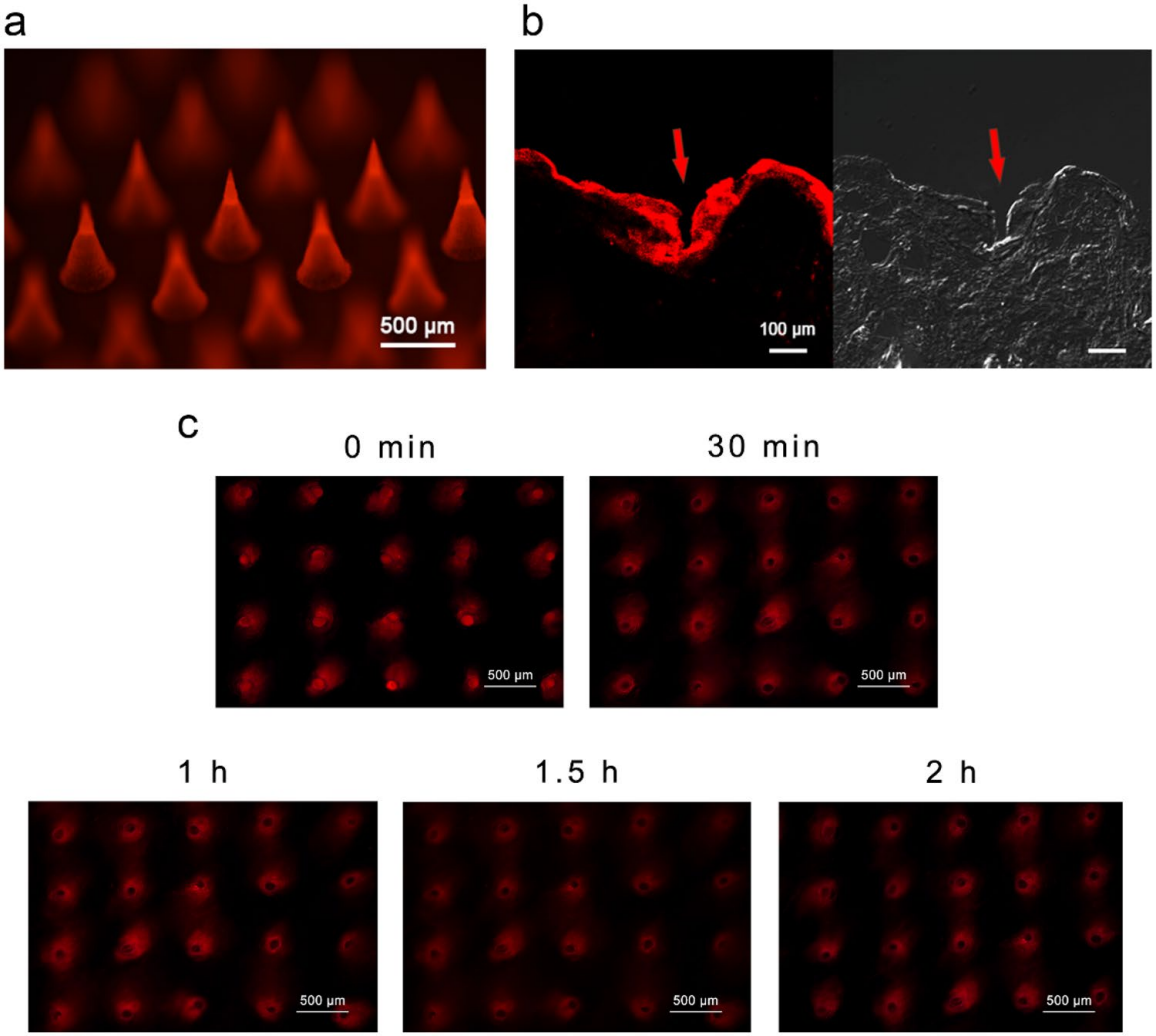
Skin insertion of SRB-loaded microneedles *in vitro*. (a) Fluorescence microscopy of SRB-loaded MN. (b) Fluorescence and bright-field microscopy of a longitudinal section of skin tissue of rats 2 min after SRB-loaded MNs pierce the skin. (c) Diffusion of SRB after skin insertion at 0, 30, 60, 90, and 120 min. Abbreviations: SRB, sulforhodamine B; MN, microneedle.

To assess MN insertion into the skin and determine the distribution of loaded substances released from MNs, SRB-loaded MNs were inserted into the dorsal skin of rats. The bright-field and fluorescence microscopic images of the skin sections showed that MNs penetrated the epidermis, and the fluorescent dye diffused in the dermis layer (approximately 100 μm or deeper) and surrounding area (Figure 3b).

The fluorescence microscopic images of the skin surface show the SRB diffusion state in the skin at different time intervals (Figure 3c). With the dissolving of the MN, the loaded dye was released into the skin, and red fluorescent microconduits were formed. Within 2 h, the dye spread from the penetration site to the surrounding tissues. With the diffusion of SRB, the fluorescence intensity in the channel pores diminished, which was consistent with the diffusion of SRB into the deeper layer of the skin (Figure 3b).

### Skin primary irritation test after colchicine-loaded microneedle application

**Figure 4.**
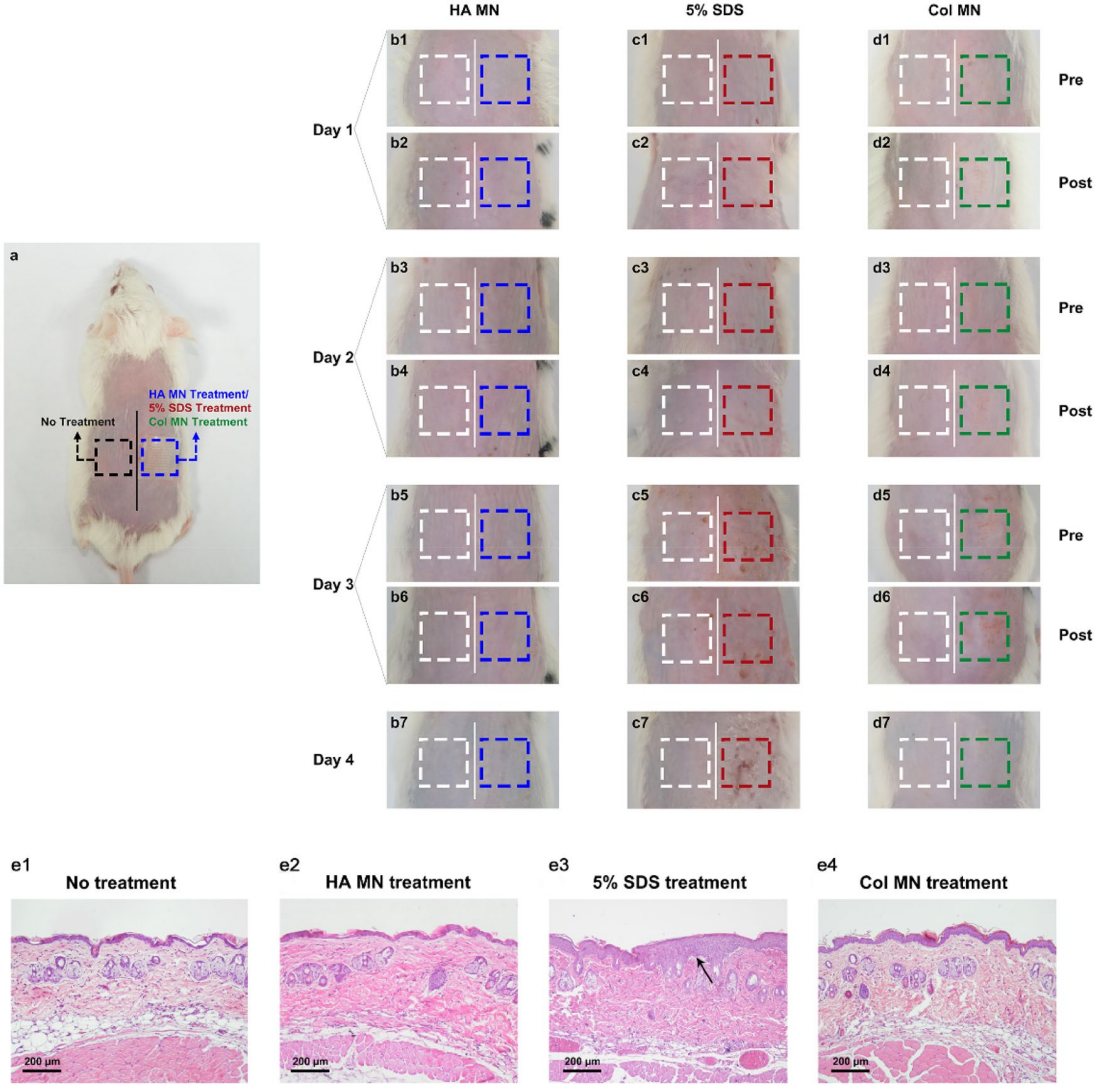
Skin irritation test after different treatments. (a) Diagram of the experimental design showing the skin treatments as annotated. (b–d) Illustration of the dorsal skin of rats after blank MN (b), 5% SDS (c), or Col-loaded MN (d) treatment. (e) Representative H&E staining images of the skin sections of each group. Abbreviations: MN, microneedle; Col, colchicine; SDS, sodium dodecyl sulfate; H&E, hematoxylin and eosin; *n* = 4.

Skin erythema and edema were evaluated using a self-controlled skin irritation test for 3 days (Figure 4a). Similar to the untreated dorsal skin area on the left side of the midline, there was no skin irritation after treatment with blank MNs (Figure 4b). However, the SDS group showed redness, swelling, and eschar, which lasted for more than 24 h (Figure 4c), while in the Col-loaded MN group, irritation could be hardly observed; even if there was slight erythema, it recovered within 24 h (Figure 4d).

Histological sections of treated skin are shown and compared with those of untreated skin and SDS-treated skin in Figure 4(e). No inflammatory cell infiltration or thickened epidermis was observed after the repeated application of blank MNs and Col-loaded MNs, indicating minimal skin irritation, including following longer-term irritation tests (Figure S6).

### *In vitro* skin permeation and *in vivo* pharmacokinetics studies of col-loaded MN

**Figure 5.**
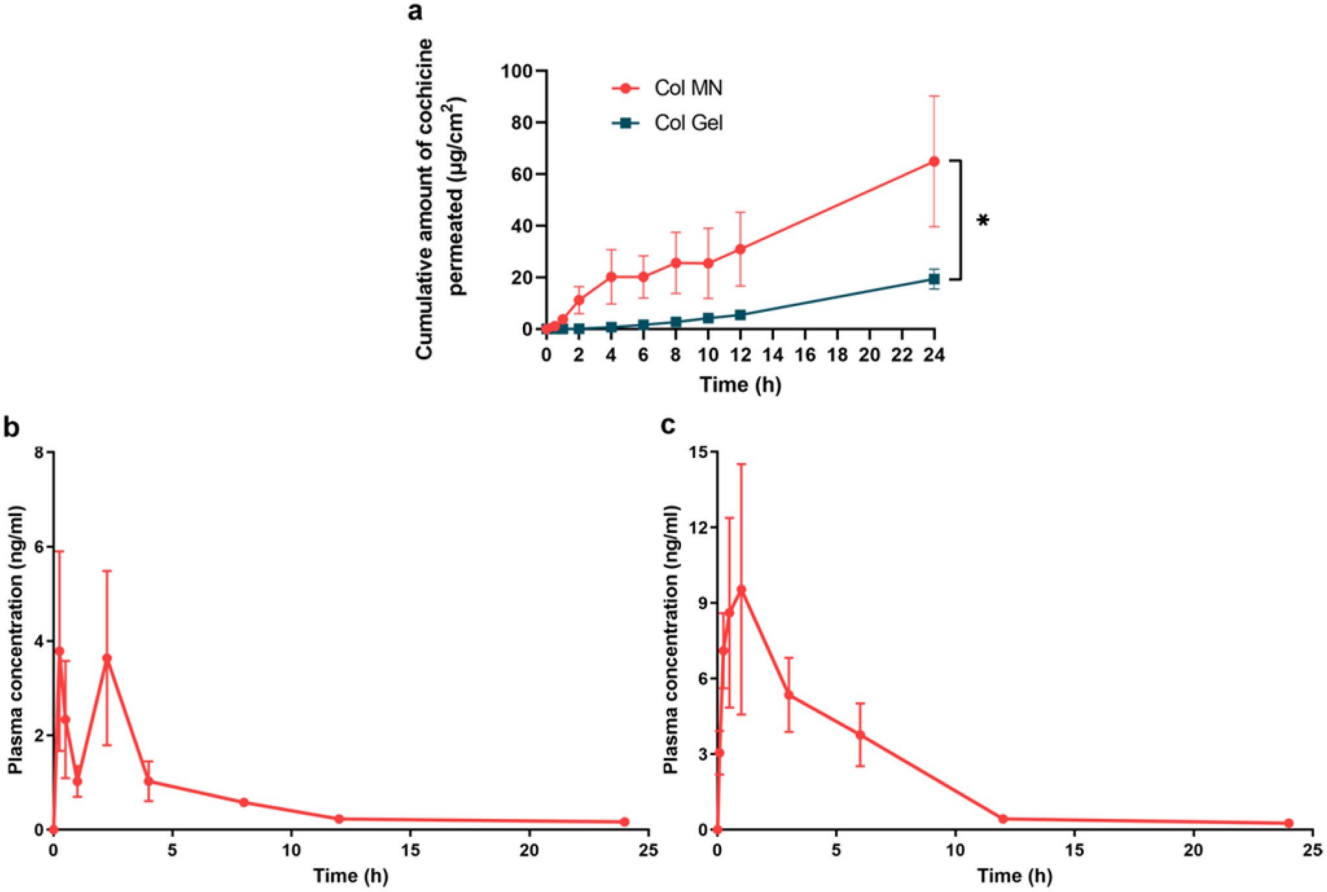
Skin permeation *in vitro* and pharmacokinetics *in vivo* of colchicine-loaded microneedles. (a) Cumulative Col permeate from Col-L gel and Col-loaded MN *in vitro*. * indicates statistical significance at *p*  < .05; *n* = 3. Changes in plasma Col concentration in rats with time within 24 h after intragastric administration of Col solution (b) and dermal application of Col-loaded MN (c); *n* = 4. Abbreviations: Col, colchicine; MN, microneedle.

The 24 h diffusion curve showed that Col that permeated in the Col-L gel group accumulated slowly, to 19.35 ± 3.88 μg after 24 h; Col permeated 3.36-fold more rapidly in the Col-loaded MN group, to 64.98 ± 25.38 μg after 24 h (*p*  < .05) (Figure 5a).

Col-loaded MNs and Col solution (*i.g.*) were administered to rats to comparatively assess Col pharmacokinetics. The Col gavage group showed double peaks at 0.25 h and 2.25 h, may indicate that hepatoenteric circulation occurred, and the Col plasma concentration remained above 0.58 ± 0.09 ng/mL for 8 h (Figure 5b); plasma concentrations of Col in the MN group increased to a peak (*C_max_*) of 9.54 ± 4.97 ng/mL at 1 h (*T_max_*) (Figure 5c). After that, the Col concentration slowly decreased and remained above 0.43 ± 0.06 ng/mL for 12 h, which was 50% longer than that in the Col solution group.

The area under the curve (AUC) in the gavage group was 16.27 ± 4.60 ng·h/mL, whereas that in the Col-loaded MN group was 52.77 ± 14.45 ng·h/mL. The relative bioavailability of Col-loaded MN compared to Col solution gavage was 58.13%.

### Edema recovery of colchicine-loaded microneedle in rats with acute gout

**Figure 6.**
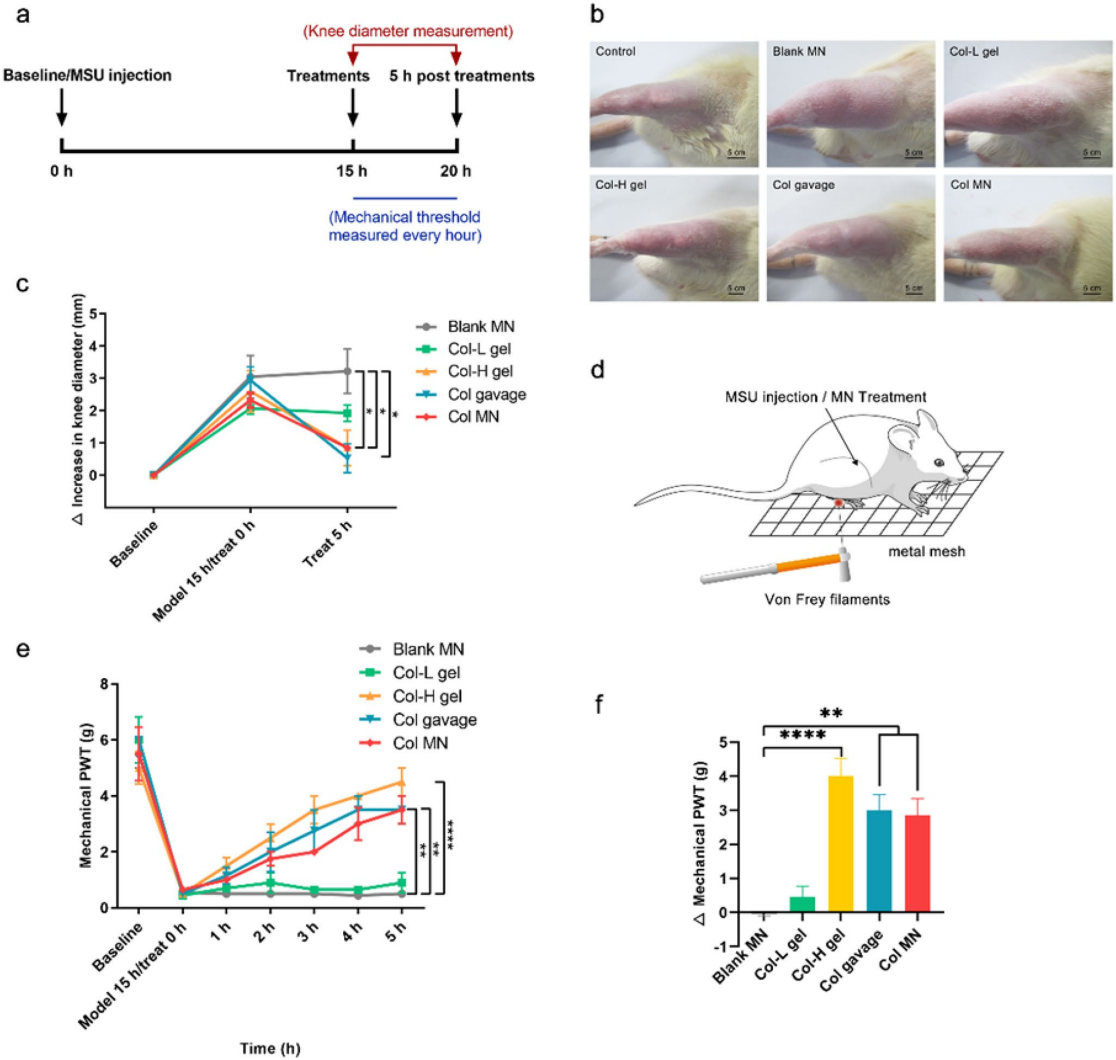
Comparison of pharmacodynamics in different Col administration groups. (a) Regime of acute gout modeling in rats and pharmacodynamic evaluation; the knee joint edema was assessed at 0 and 5 h post-treatments, during which the hind paw mechanical pain threshold of rats was measured every hour. (b) Photos of the knee joints of acute gout model rats 5 h after different administration strategies. (c) Changes in diameters of the knee joint; the first point on the abscissa represents the beginning of modeling (baseline), the second point represents the beginning of treatments 15 h after modeling, and the third point represents that the treatments were applied for 5 h. (d) Schematic diagram of von Frey filament application. (e) Changes in the mechanical PWT of the hind paw with time after modeling and treatments. (f) Difference of PWT pretreatments and 5 h after treatments. *, **, and **** indicate statistical significance at *p*  <  .05, *p*  <  .01, and *p* <  .0001. Abbreviations: Col, colchicine; MN, microneedle; MSU, monosodium urate; PWT, paw withdrawal threshold; *n* = 4.

The knee joint edema was assessed at baseline, 15 h post-MSU injection, and 5 h post-treatments (Figure 6a). Fifteen hours after modeling, the joint showed swelling, and the knee diameter increased by approximately 2.60 mm (Figure 6b). Five hours after treatment, the blank MN and Col-L gel groups still showed joint swelling and a slight increase in knee diameter. The Col-H gel, Col gavage, and Col-loaded MN groups showed relief of joint swelling, and their joint diameters were significantly shorter than those of the blank MN group (*p*  <  .05) (Figure 6c).

### Mechanical hypernociception relief of colchicine-loaded microneedles

To assess the effectiveness of different treatments, the ability of rats to withstand mechanical stimulation was measured in the hind paws (Figure 6a, d). The trend in PWT change was consistent with that of the knee joint diameter. Specifically, the blank MN group exhibited no recovery in threshold (Figure 6e). The maximum difference value in PWT before and after treatment was −0.55 ± 0.05 g, indicating that the MN matrix material does not alleviate acute gout inflammation (Figure 6f). The Col-L gel group showed no therapeutic effect with the same dose, and the difference was 0.45 ± 0.032 g. The PWTs in the Col gavage and Col-loaded MN groups recovered to 50% and 52% of the baseline, respectively, and these rats recovered significantly better than those in the blank MN group (*p*  <  .01). However, the anti-hyperalgesic effect of the gavage group began to subside after 4 h. Although the PWTs in the Col-H gel group were restored to 80% 5 h after Col-H administration, the dosage of Col was 4.35-fold greater than that in the Col-loaded MN group. Thus, Col-loaded MNs alleviated mechanical allodynia more effectively than Col-H and Col gavage.

## Discussion

In this study, dissolvable Col-loaded MNs, mechanically forming micropores and releasing Col into the skin, significantly enhanced transdermal permeability and addressed the limitations of its oral administration (Cheung et al., 2014). Moreover, compared with injection and oral routes, MNs possess the characteristic advantages of low cost, minimal invasion, easy self-administration, and first-pass avoidance (Serhan et al., 2004; Yang et al., 2021).

The characterization results suggested that MNs had a uniform shape, and the loaded substances were more concentrated on the tip of the needles. Dehydrating the HA solution results in the formation of a polymer network, which locks the water molecules *via* polarity and hydrogen bonds, and concentrates the drug on the needle tip (Nijenhuis et al., 2008; Liu et al., 2012; Chen et al., 2018). Owing to the conical geometry of the MN and elastic characteristics of the skin, approximately one third to one half of the MN body dissolves and diffuses into the skin (Li et al., 2019). Therefore, the transdermal delivery efficiency of MNs can be improved by loading the drug on the tips, even if the MN is only partially inserted into the skin. Furthermore, the study of Col stability in the Col-loaded MN array over time and at a high temperature (Figures S2 and S3) verified that there was no reaction between HA and Col.

We also assessed whether the Col-loaded MNs could pierce the skin effectively. It has been reported that the force required for skin penetration by an MN is 0.058 N/needle (Liu et al., 2014). The maximal bearing force of MNs (HA MW: 340 kDa) tested was 0.289 ± 0.019 N/needle, and the pressure applied by a human thumb is generally above 28.1 N, which ensures effective skin penetration of the 100-MN array without breaking. Results also indicated that MNs dissolved completely within 30 min of application, showing the expected drug delivery performance (Figure S4).

For transdermal drug delivery, skin biosafety is necessary to be considered. In the skin irritation test, compared with the positive control of 5% SDS, the Col-loaded MN was well tolerated by mice. The slight erythema might result from a vasodilatation response to the physical compression during MN application, and might be more serious than that induced in human skin (Yang et al., 2020).

In the *in vitro* transdermal permeation experiment, the Col-loaded MN group showed a higher diffusion rate than the Col-L gel group. The pharmacokinetic assay indicated that Col loaded in MNs was quickly released into the blood. The relative and absolute bioavailability (Figure S7) of the Col-loaded MN array implied that Col might have partially remained in the skin tissue, resulting in relatively low blood concentration, which is consistent with previous studies (Schulz et al., 2020). Additionally, the local concentration of Col may have been higher than that in the circulating blood; this implies therapeutic benefits without potential systemic side effects, as Col in systematic circulation has an inhibitory effect on the mitosis of body cells; in addition, high Col blood concentration is not desired in clinical contexts (Nasr et al., 2020). Compared with the results of previous studies on ethosomes and cubosomes, Col-loaded MNs had faster permeation and more stable blood concentration (Roddy & Mallen, 2017; Zhang et al., 2019).

The present study has some limitations. It should be noted that the Col-loaded MN has an initial burst of Col release in 0.25–3 h. To avoid possible side effects and toxic reactions, the formulation of dissolving MNs should be further optimized with HA and other biocompatible polymers for more sustainable drug release, or more precise drug delivery profile under different clinical conditions (Roddy & Mallen, 2017; Nasr et al., 2020). As studies were conducted on rats, clinical pharmacokinetic trials should be performed for dose optimization in future. In this case, the improvement of the shape of the needle body may increase the succeed rate and depth of penetration of the microneedle, and increase the utilization rate of the drug contained in the microneedle patch (Aoyagi et al., 2008). Another approach to improve the compliance of patients with gout is to develop an instantly detachable MN. The drug-containing needle tips can be left within the skin shortly after patch application, while the base panel can be removed to avoid drug loss or patch shedding by friction (Lee et al., 2021). In addition, future studies need to be carried out using human skin *in vitro* and *in vivo* before the successful transition to clinical use.

## Conclusions

The results of this study indicate that dissolvable Col-loaded MN topically pierces the skin with little irritation, rapidly releases 3.36-fold more Col compared with gel form, and exhibits significant anti-inflammatory effects equivalent to those of the oral route in acute gout model rats over 5 h. These results offer the possibility of rapid relief from gout attacks, reduced gastrointestinal side effects of Col, and improved patient compliance in the future.

## Supplementary Material

Supplemental Material
