# Supplementary material for: Transdermal delivery of colchicine using dissolvable microneedle arrays for the treatment of acute gout in a rat model
Source: Drug Deliv. 2022 Sep 13;29(1):2984–94. doi: 10.1080/10717544.2022.2122632 (PMC9487926; doi:10.1080/10717544.2022.2122632)
Supplement: Supplemental Material [file IDRD_A_2122632_SM6567.docx]

# Supplementary Material

## Transdermal delivery of colchicine using dissolvable microneedle arrays for the treatment of acute gout in a rat model

### S1. HPLC condition

HPLC: Chromatographic separations were carried out on an Agilent ZORBAX SB-C8 column (Santa Clara, CA, USA) at 25 °C, using acetonitrile: water (30: 70, v/v) as the mobile phase. The flow rate was set at 0.6 mL/min and the injection volume was 10 μL. The detection wavelength was 353 nm.

According to the HPLC results, the retention time of Col was 2.9 min. The linearity was good at 0–1.0 mg/mL and the linear equation was Y = 299.4X − 1.227 (R^2^ = 0.9998) (Figure S1).


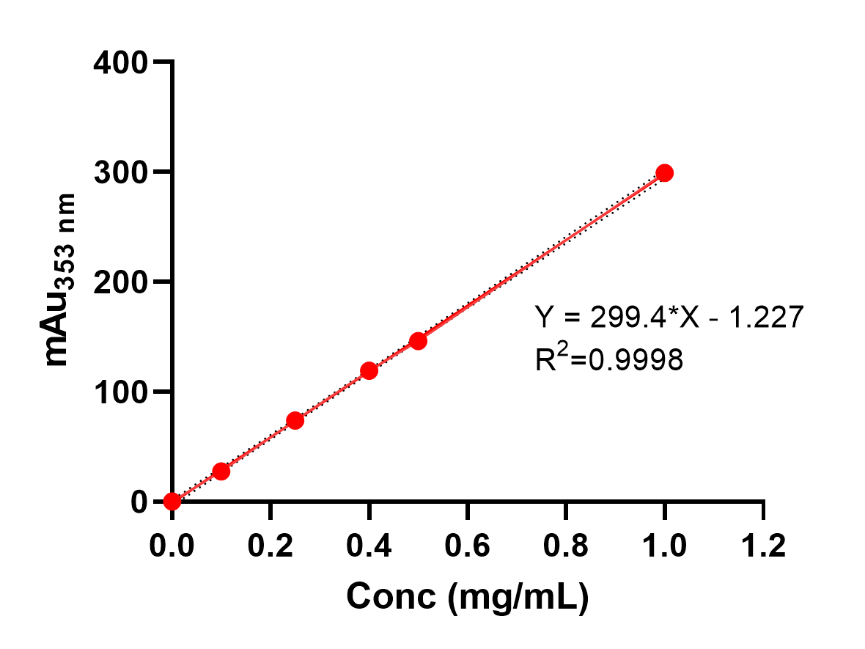


**Figure S1**. Standard curve of colchicine determined using HPLC.

### S2. LC-MS/MS condition

LC-MS/MS: The analytical column consisted of Welch Ultimate XB-C18 (2.1 × 100 mm, 3 μm) with a start temperature of 40 °C. Mobile phase A was 0.1% formic acid and B was acetonitrile. The flow rate was 0.4 mL/min, the sample volume was 2 μL, and the column temperature was 20 °C. The ion source involved an electrospray ion source in positive ion mode. The MS parameters of Col were a collision energy of 35±15 eV and gas temperature of 550 °C.

According to the LC-MS results, the retention time of Col was 1.7 min. The linearity was good at 0–12.5 ng/mL and the linear equation was Y= 1.70318e^6^ x + 418.95234 (R^2^ = 0.99992) (Figure S2).


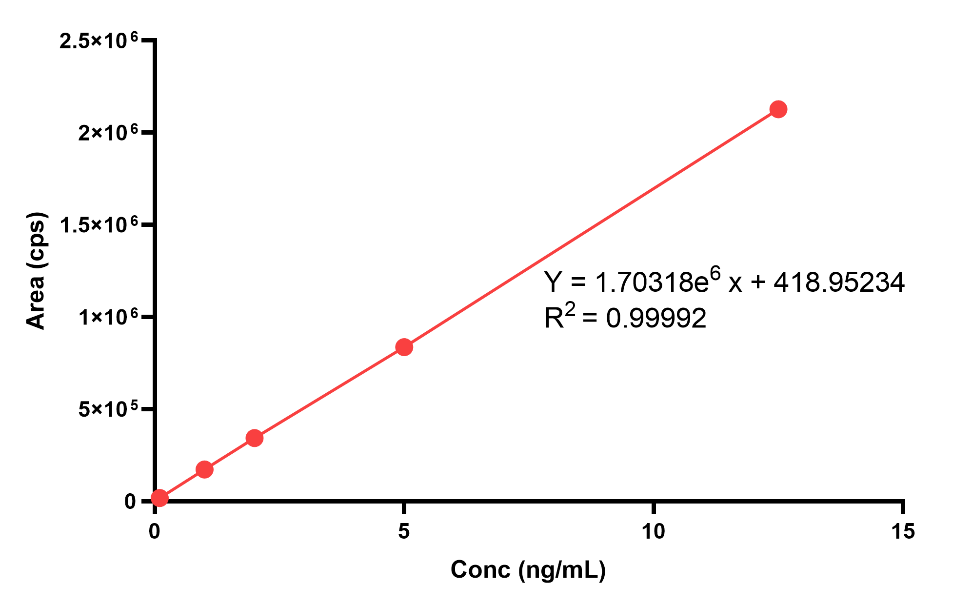


**Figure S2**. Standard curve of colchicine determined using LC-MS.

### S3. Temperature stability of Col-loaded MN

Three batches of Col MNs (n = 4) were stored in sealed bags at 4 °C, room temperature (25±2 °C), and 40 °C for 1 month. Subsequently, MNs were dissolved in 1 mL of 1× PBS, shaken at 100 rpm for 2 h, vortexed to obtain a uniform solution, and filtered through a 0.22-μm filter. The filtrate was measured by HPLC at 353 nm to determine the content of Col in the MNs.

The Col content of MNs was determined after storage at 4 °C, 25 °C, and 45 °C for 1 month (Figure S2). Col retention at 45 °C was lowest, with an average of 167.9±12.08 µg. The amount of loaded Col retained in MNs at 4 °C also decreased, with an average of 198.5±4.40 µg, which was significantly different from the baseline (*p* < 0.0001). There was no statistical difference from the baseline at 25 °C, with an average value of 262.2±4.95 µg. These results suggest that Col-loaded MNs are stable at 25 °C for at least 1 month, possibly because of their solid-state characteristics.


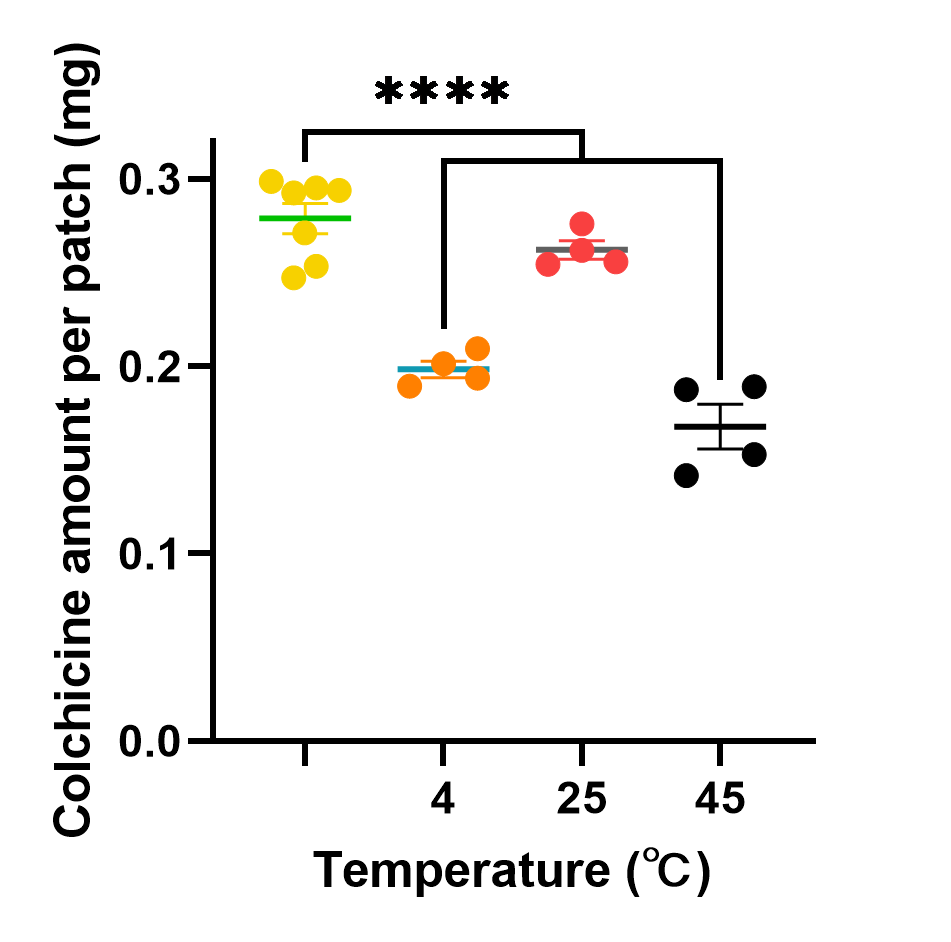


**Figure S3**. Amount of Col in the 10 × 10 Col-loaded MN after 1 month of storage at 4 °C (refrigerator temperature), 25 °C (normal temperature), or 45 °C (accelerating temperature). **** indicates statistical significance at *p* <0.0001.

### S4. Hygroscopy of Col-loaded MN

The same batch (n = 4) of Col MNs was stored in a cup of saturated sodium chloride solution at 25 °C at a relative humidity of 75%. The morphology of the MNs was observed on days 0, 10, and 20. The weight of the MNs was measured on days 0 and 20, and the weight change was calculated. Then, the content of Col in the MNs was measured using HPLC.

To evaluate the hygroscopicity of Col-loaded MNs, MNs were stored at 25 °C at a relative humidity of 75% for 20 days (Figure S3). The overall height did not change significantly (Figure S3a). After 20 days, the weight of a single 10 × 10 MN patch increased by 7.42%, and the average Col amount was 276±18.44 µg, which was close to the amount measured immediately after preparation (Figure S3b and S3c). The results indicate that although Col MN has a certain hygroscopicity at high humidity, the amount of Col remains almost unchanged.


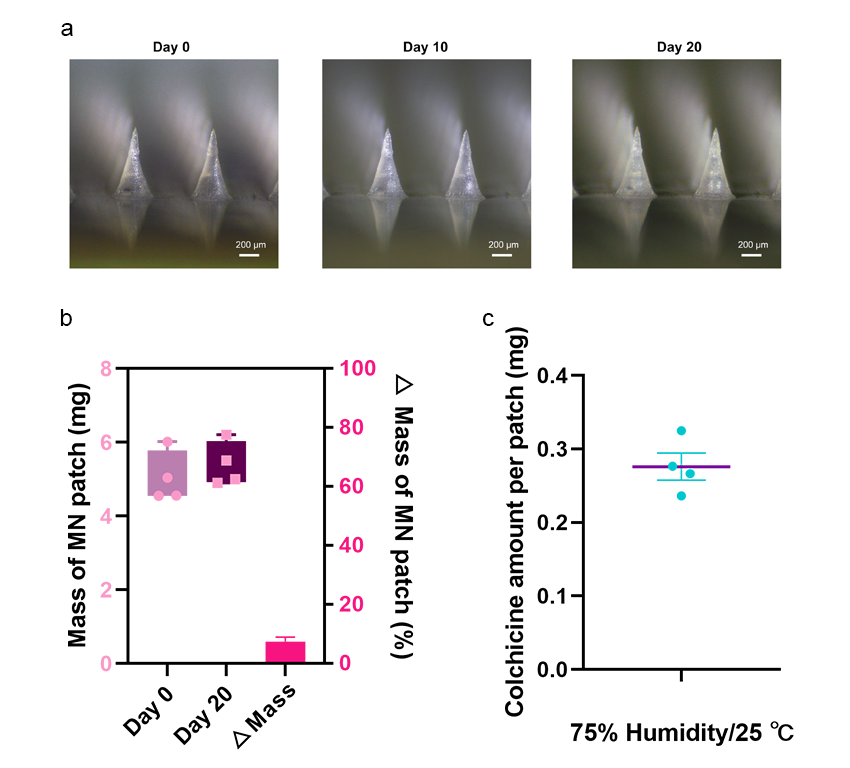


**Figure S4.** Hygroscopy measurement of 10 × 10 Col-loaded MN. (a) Stereomicrographs of Col MN on days 0, 10, and 20. (b) Comparison of the mass changes of Col MN on days 0 and 20. (c) Col amount per Col MN array on day 20.

### S5. Dissolving study of Col-loaded MN

To evaluate the dissolution velocity of Col MNs in the skin, Col MNs were applied to the shaved dorsal skin of rats and removed 30, 60, 90, and 120 min after the initial application. Therefore, the dissolution of the MNs was examined and imaged using a stereomicroscope (Figure S4a).


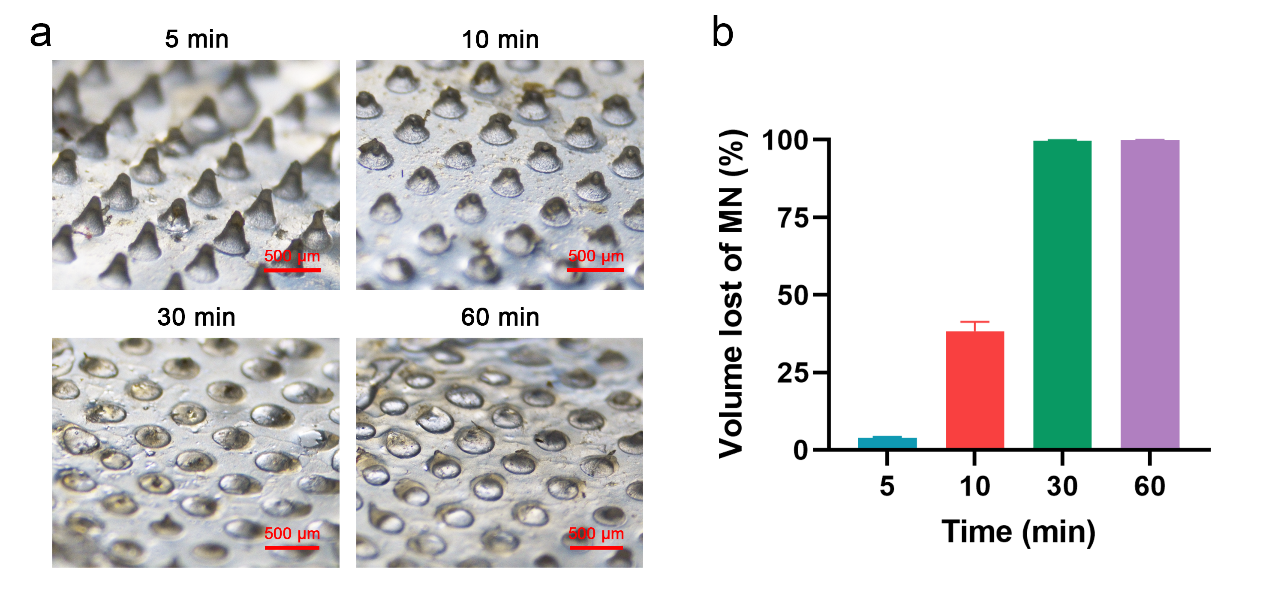


**Figure S5.** Dissolution of Col MN. (a) Illustration of MN 5, 10, 30, and 60 min after application to the skin of rats. (b) Reduction of MN volume with time.

When removing the Col MNs, no visible damage to the MN array was observed. After piercing the skin for 5 min, the height of the needle body was reduced from 600 μm to approximately 400 μm; approximately 33% of the original length was lost, equivalent to a 3.7% reduction in the volume of MNs (Figure S4b). Smooth residue and a round top were observed owing to the cone geometry of the needle bodies. After 10 min, the height decreased from 600 to 150–180 μm. The upper 420–450 μm of the MN completely dissolved; almost 70–75% of the length and 34–42% of the volume was lost, and the needle tip become blunt and shorter. Furthermore, the needle bodies of MNs were 100% dissolved 30 and 60 min after application; thus, the MN could achieve complete drug release for at most 30 min after application.

When an MN fails to penetrate the skin, it lies on the skin surface, exhibits a bent shape, and is unable to dissolve [1]. The reduction in the height of the MN in the dissolution experiment showed that the MN successfully penetrated the skin and dissolved. Indeed, the drug release rate mainly depends on the dissolution rate of the MN. Owing to the high hydrophilicity of HA, the MN completely dissolved within 30 min after application [2, 3].

### S6. Skin irritation test after long-term repetitive application of Col MN


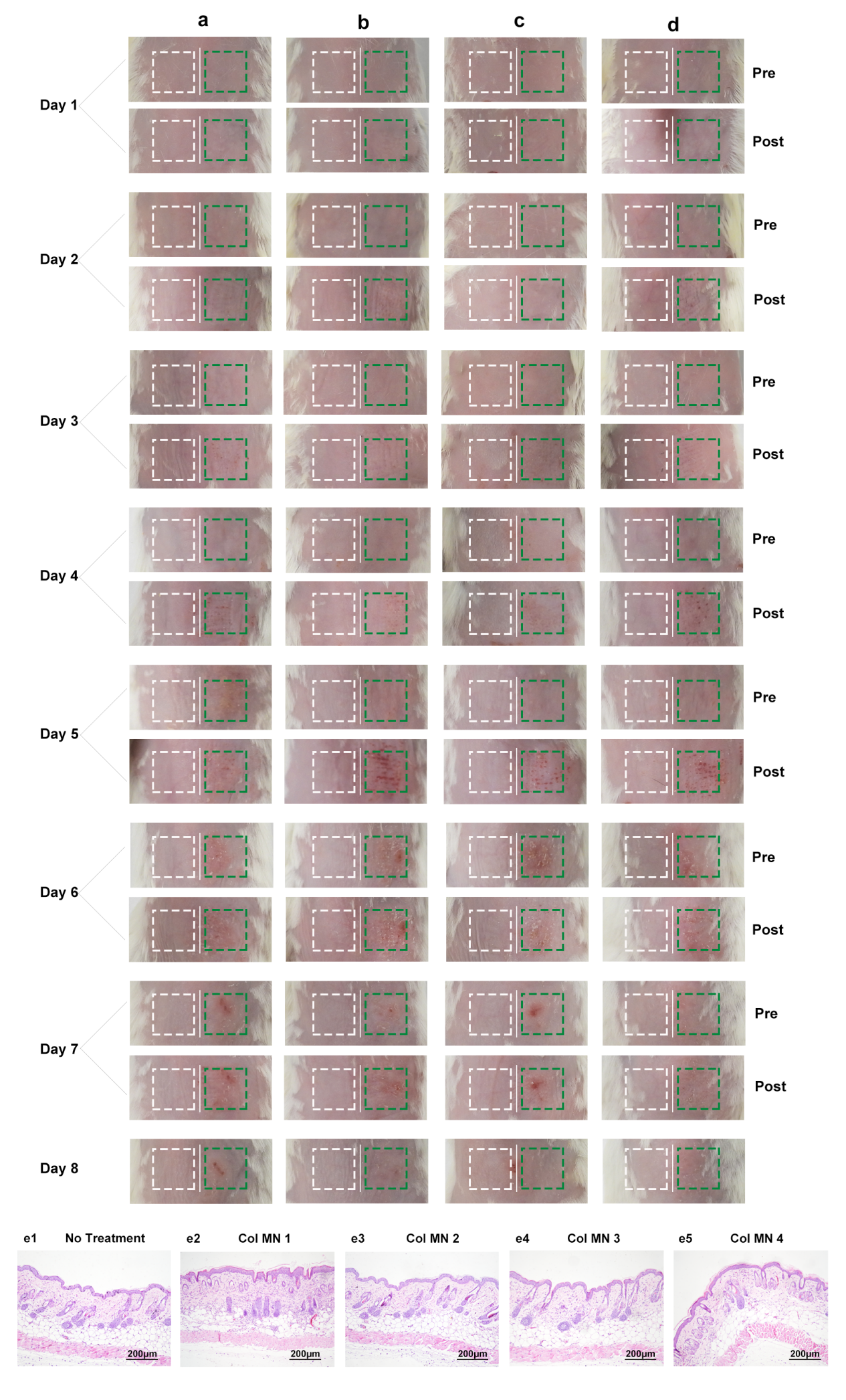


**Figure S6.** Skin irritation test after application of Col MN. (a-d) Dorsal skin of rats after Col-loaded MN treatment; green box on the right is the MN application area, and symmetrical white box on the left is the no treatment skin area. (e) Representative H&E staining images of the skin sections of each mouse.

To verify the irritation of the skin by repeatedly applying Col MN, we conducted an 8-day skin irritation experiment, in which a colchicine-loaded microneedle was applied to the dorsal surface of mouse each day for seven successive days, and H&E-stained sections were prepared on day 8. According to the results, when compared with the untreated dorsal skin area on the left side of the midline, long-term repetitive application of colchicine-loaded microneedles also causes little irritation and recovery could occur quickly.

### S7. *In vivo* pharmacokinetics study of intravenous injection of Col solution


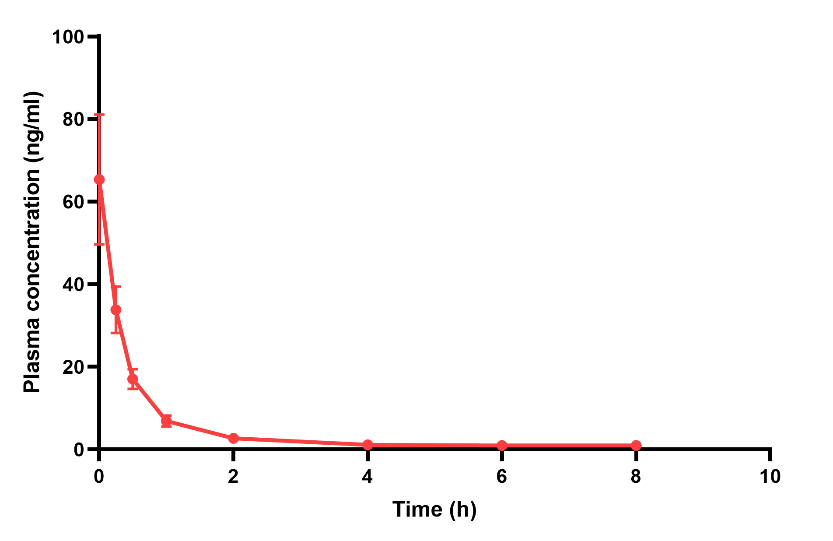


**Figure S7**. Changes in plasma Col concentration in rats with time within 8 h after intravenous administration of Col solution; n = 4.

To evaluate the absolute bioavailability of Col-loaded MNs, we performed an intravenous injection of 1mL of Col (0.05 mg/mL) normal saline solution to each rat and analyzed the changes of blood concentration in rats plasma within 8h. The absolute bioavailability was calculated using the following formula (2).

F_abs_ = $\frac{\text{AUC}\text{T}\text{×D}\text{iv}}{\text{AUC}\text{iv}\text{×D}\text{T}}$ (2)

The area under the curve (AUC) was 37.48 ± 2.748 ng·h/mL. The absolute bioavailability of Col-loaded MN was 25.23%, which corresponded to relative bioavailability. High Col blood concentration is not desired due to the potential systemic side effect.

### References

[1] H. Yang, G. Kang, M. Jang, D.J. Um, J. Shin, H. Kim, J. Hong, H. Jung, H. Ahn, S. Gong, C. Lee, U.W. Jung, H. Jung, Development of Lidocaine-Loaded Dissolving Microneedle for Rapid and Efficient Local Anesthesia, Pharmaceutics 12(11) (2020) 1067. DOI: 10.3390/pharmaceutics12111067.

[2] B.Z. Chen, M. Ashfaq, X.P. Zhang, J.N. Zhang, X.D. Guo, In vitro and in vivo assessment of polymer microneedles for controlled transdermal drug delivery, J Drug Target 26(8) (2018) 720-729. DOI: 10.1080/1061186X.2018.1424859.

[3] J. Cao, N. Zhang, Z. Wang, J. Su, J. Yang, J. Han, Y. Zhao, Microneedle-Assisted Transdermal Delivery of Etanercept for Rheumatoid Arthritis Treatment, Pharmaceutics 11(5) (2019) 235. DOI: 10.3390/pharmaceutics11050235.
